# Brain Density Clustering Analysis: A New Approach to Brain Functional Dynamics

**DOI:** 10.3389/fnins.2021.621716

**Published:** 2021-04-13

**Authors:** Ashkan Faghiri, Eswar Damaraju, Aysenil Belger, Judith M. Ford, Daniel Mathalon, Sarah McEwen, Bryon Mueller, Godfrey Pearlson, Adrian Preda, Jessica A. Turner, Jatin G. Vaidya, Theodorus Van Erp, Vince D. Calhoun

**Affiliations:** ^1^The Tri-Institutional Center for Translational Research in Neuroimaging and Data Science, Georgia Institute of Technology, Georgia State University, Emory University, Atlanta, GA, United States; ^2^School of Electrical and Computer Engineering, Georgia Institute of Technology, Atlanta, GA, United States; ^3^Department of Psychiatry, The University of North Carolina, Chapel Hill, Chapel Hill, NC, United States; ^4^Department of Psychiatry, University of California, San Francisco, San Francisco, CA, United States; ^5^San Francisco VA Medical Center, San Francisco, CA, United States; ^6^Department of Psychiatry and Biobehavioral Sciences, University of California, Los Angeles, Los Angeles, CA, United States; ^7^Department of Psychiatry, University of Minnesota, Minneapolis, MN, United States; ^8^School of Medicine, Yale University, New Haven, CT, United States; ^9^Department of Psychiatry and Human Behavior, University of California, Irvine, Irvine, CA, United States; ^10^Department of Psychology, Georgia State University, Atlanta, GA, United States; ^11^Department of Psychiatry, The University of Iowa, Iowa, IA, United States

**Keywords:** functional magnetic resonance imaging, brain dynamics, independent component analyses, resting state– fMRI, density clustering

## Abstract

**Background:**

A number of studies in recent years have explored whole-brain dynamic connectivity using pairwise approaches. There has been less focus on trying to analyze brain dynamics in higher dimensions over time.

**Methods:**

We introduce a new approach that analyzes time series trajectories to identify high traffic nodes in a high dimensional space. First, functional magnetic resonance imaging (fMRI) data are decomposed using spatial ICA to a set of maps and their associated time series. Next, density is calculated for each time point and high-density points are clustered to identify a small set of high traffic nodes. We validated our method using simulations and then implemented it on a real data set.

**Results:**

We present a novel approach that captures dynamics within a high dimensional space and also does not use any windowing in contrast to many existing approaches. The approach enables one to characterize and study the time series in a potentially high dimensional space, rather than looking at each component pair separately. Our results show that schizophrenia patients have a lower dynamism compared to healthy controls. In addition, we find patients spend more time in nodes associated with the default mode network and less time in components strongly correlated with auditory and sensorimotor regions. Interestingly, we also found that subjects oscillate between state pairs that show opposite spatial maps, suggesting an oscillatory pattern.

**Conclusion:**

Our proposed method provides a novel approach to analyze the data in its native high dimensional space and can possibly provide new information that is undetectable using other methods.

## Introduction

Recent work in fMRI has focused on relaxing the assumption that the brain is static during an experimental session. There are many studies that have shown that the brain is time-varying (or dynamic) within a single scanning session (Chang and Glover, 2010; Sakoglu et al., 2010; Hutchison et al., 2013; Calhoun et al., 2014; Faghiri et al., 2018; Lurie et al., 2020). One common way to analyze the dynamic aspect of the brain is by estimating time varying connectivity using sliding window paired with a connectivity estimator such as Pearson correlation (Handwerker et al., 2012; Allen et al., 2014). This approach is useful and widely used due in part to its simplicity, but it has some limitations. Windowing the data results in smoothing the temporal information in fMRI, potentially missing important information. A more minor issue with this method is that one has to use a specific window length for this analysis and changing this window length can change the final results (Sakoglu et al., 2010; Shakil et al., 2016). To remedy the smoothing problem several methods have been proposed that are either more instantaneous (Shine et al., 2015; Omidvarnia et al., 2016; Faghiri et al., 2020) or use different filtering and time-frequency approaches to explore the full spectrum of connectivity (Chang and Glover, 2010; Yaesoubi et al., 2015; Faghiri et al., 2021). For more detailed reviews of time-varying connectivity please (see Bolton et al., 2020; Iraji et al., 2020a). Many of these proposed connectivity-based approaches do not directly leverage the dynamics of the data in its original high dimensional space (i.e., the data is used to calculate the sliding window correlation, which is calculated between each of the component pairs separately). This causes the data to be examined in many two-dimensional (2D) spaces independent of other 2D spaces (where each 2D space is specific to a component pair). Recently novel methods have been proposed that try to go from these 2D spaces to higher dimensions using different methods (Faskowitz et al., 2020; Iraji et al., 2020b).

Apart from connectivity-based approaches, there are others that aim to extract dynamism directly from activity domain information. For example, hidden Markov models have been used to estimate several hidden states from the activity data in fMRI (Karahanoğlu and Van De Ville, 2017; Vidaurre et al., 2018). Others have either including activity information in the pipeline directly (Fu et al., 2021) or have instead focused on a metric calculated based on activity like power (Chen et al., 2018). In addition, there are a family of methods based on co-activation between different part of the brain that directly include activity information in their analysis pipeline too (Liu and Duyn, 2013; Karahanoglu and Van De Ville, 2015).

Over the last decade, many studies have compared the brains of individuals with schizophrenia with those of healthy controls using both resting state (Damaraju et al., 2014; Guo et al., 2014; Faghiri et al., 2021) and task fMRI (Boksman et al., 2005; Ebisch et al., 2014). Recently more emphasize has been put on methods that explore the dynamic aspects of the brain (Damaraju et al., 2014; Kottaram et al., 2019; Gifford et al., 2020; Faghiri et al., 2021). Using dynamic methods, some studies have reported lower dynamism in individuals with schizophrenia (Miller et al., 2016; Kottaram et al., 2019; Gifford et al., 2020) or in individuals with high risk for schizophrenia (Mennigen et al., 2018a). In addition, Rashid et al. (2016) showed that using dynamic connectivity instead of static connectivity we can reach better classification of individuals with schizophrenia and bipolar disorder. For a detailed review on the matter (see Mwansisya et al., 2017; Mennigen et al., 2019).

In this study, we propose a novel approach to study brain dynamics in resting state fMRI. We consider the brain data at each time point as a location in a high dimensional space defined by multiple time series. Analyzing brain data within a high dimensional temporal space allows us to consider the fMRI data for each subject as a path along which each individual’s brain is moving within this high dimension space. In contrast to pairwise connectivity-based methods, we move beyond the bivariate/2D space (i.e., a focus on two time series without considering other time series) and work in a high dimensional space. Our proposed method does not use any temporal windowing unlike many sliding window methods (e.g., sliding window Pearson correlation) or filtering (e.g., amplitude of low-frequency fluctuation) therefore allowing us to use the information from the whole spectrum of data. In addition, unlike methods based on co-activation, our method is not sensitive to the absolute value of the time series and instead captures the density around each sample in a high dimensional space.

In the next section, we describe our proposed method in detail, show its utility via simulation data, and implement it on a real-world data set to compare the patients with schizophrenia (SZ) to healthy control (HC) participants.

## Materials and Methods

### Calculating Density

Let us assume our data for each subject includes C time series (each representing a separate component) with T time points. In our framework, this would mean that each subject occupies one location in a space with C dimension at each of the time points (i.e., the location of subject j at time t in this high dimension space is *x*_*j,t*_). This is a vector with a length of C.

Using these vectors, a scalar metric called density is defined (*d*_*j,t*_). This metric is higher if *x*_*j,t*_ has several close points in our defined space. The formula for calculating*d_j,t_* is:

(1) $$d_{j,t}=\frac{1}{\sum_{t_{0}\in {\text{N}}_{j,t}}\sqrt{{||x_{j,t}-x_{j,t_{0}}||}_{2}}}$$

N_*j*,*t*_is a neighborhood around *x*_*j,t*_ defined by a number of closest points to *x*_*j,t*_. So essentially, we calculate the distance between *x*_*j,t*_ and all *x*_*j,t_0*_ for all values of *t_0* and pick the ones that have the small distance value. We call this parameter the city size and it essentially determines the size of detectable high-density neighborhoods (hence the name city size). Note that we have one density time series with length T for each subject. i.e., density is defined using all components time series for each subject.

Next, we define a density threshold for each subject based on all of the density values for that specific subject:

(2) $$d\,\_\,t\,h\,r_{j}=\max_{\forall t}\left(d_{j,t}\right)\times c\,u\,t\,o\,f\,f$$

**FIGURE 1:**
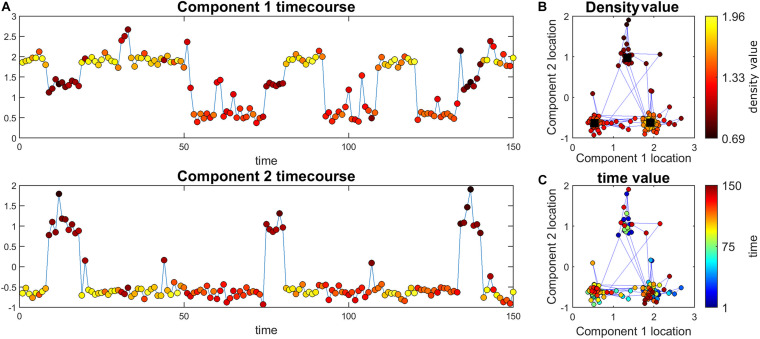
Illustration of the main ideas of the proposed method. **(A)** Simulated time course for the two components. The color of markers represents the density value of the data at each point. For each time point, density measures the closeness of that time point to all other time points. Note that this value is the same for the two components at each point (i.e., each point has an density value). **(B)** The black squares are the true high traffic nodes. The blue lines connect points that are adjacent in time. The color of points shows the density value. Here, the points close to high traffic nodes have higher density. **(C)** The same information as part b but the color of the points represent time.

Where max_∀*t*_(*d*_*j*,*t*_) is the maximum value of all *d*_*j,t*_ for all *t* values for a specific subject (i.e., j). It is important to note that this threshold can be unique for each subject. An example value for *cutoff* can be 0.9. Next, this threshold was used to identify high-density time points for each subject (i.e., any time point with density greater than this value is defined as a high-density location (see Figure 1). Note that these high-density points are vectors with length C. Alternatively, we can calculate an average of the five highest percentile energies and use that value instead of max_∀*t*_(*d*_*j*,*t*_) to reduce the effect of outliers.

The high-density locations for all subjects were then combined and clustered using k means. The number of clusters was calculated based on the elbow criteria (Thorndike, 1953). In the last step, we used the cluster centroids estimated via k means clustering on the high-density locations to initialize a clustering of all the data for each subject. A summary of the algorithm is explained in Table 1.

**TABLE 1:** Summary of all the steps in the clustering algorithm.

Data is**x**_**N**,**T**,**C**_ where **N** is number of subjects, **T** is number of time points and **C** is number of components
1. For each subject **j** and time point **t** a. Inverse of Euclidean distance for subject **j** at time point **t** with all other time points were calculated (Euclidean distance dimension is **C**) b. The above distance vector (with length **T-1**) was sorted c. The highest subset of these **T-1** values were selected (size of this subset is determined by city size) d. These values are summed to get one value that determines the density for subject **j** at time point **t** (i.e., **d**_**j**,**t**_)
2. For each subject **j** maximum value of **d**_**j**,**t**_ for all t∈**1**:**T** was found and multiplied by a cut-off value to get a density threshold for each subject (i.e., **d**_**thr_j_**)
3. For each subject **j**, data points with density higher than **d**_**thr_j_** was selected to be used in the clustering step
4. Clustering was performed on all selected time points from all subjects.
5. The estimated clustering centroids were used to cluster the original data (i.e., **x**_**N**,**T**,**C**_).

### Simulation

To validate our proposed approach and explore the effect of different parameters of the method, we present different simulation scenarios here. All the simulations were constructed using two components for easy visualization (*C* = 2). For each simulation, we first randomly chose three locations in the 2D space to act as high-density nodes (*L*_*i*_;*i*1,2,3). A large portion of the data will be in the close neighborhood to these nodes. For each high-density node (i.e., cluster), a number is then chosen to act as that cluster size (*CS*_*i*_). Next, *CS*_*i*_ samples are drawn from a 2D Gaussian with a mean equal to *L_i* and a standard deviation equal to a parameter we call path spread. This parameter defines how closely the cluster members are spread around*L_i_*. We believe this path spread can represent the noise impact on the data. For all simulations, a number of time points are simulated to act as noise (i.e., do not belong to any high-density clusters).

Next, these simulated time series are analyzed using steps discussed the in previous section. Below, we explain different simulation scenarios that we have designed. For each simulation, we give all the parameters of the simulation (*CS*_*i*_, and path spread) and the analysis (city size, and cutoff). Note that the first two scenarios are run only once and are designed to give the readers some intuition on the reasoning behind the design of the pipeline and parameters that can impact the results.

#### Scenario 1

In this scenario, our goal was to show the effect of path spread on the analysis. For this simulation, we have used 60 for all cluster sizes (*CS*_*i*_) while we have varied the path spread of the simulation between 0.1 and 0.3. For our analysis, we used 20 as the city size and 0.9 as the cutoff for the density.

#### Scenario 2

This scenario was designed to show the effect of using a different city size for analysis. This scenario has two simulations. In the first simulation, the cluster size is constant at 60 for all clusters. Path spread was chosen as 0.1. For the analysis, 0.9 was used as cutoff value. We used variable city size between 10 and 60 for analysis. In the second simulation, cluster sizes were varied (30, 50, and 80 were chosen). All other parameters were selected equal to the previous simulation.

#### Scenario 3

For this scenario, we show that our steps used for selecting high-density time points are improving the overall method. To do this for each simulated time series, we conducted the clustering step twice; one time using all time points as data to be clustered and another time using only high-density points (our proposed approach). For each path spread, we repeated the simulations 1,000 times and, for each method, the distance between estimated cluster centroids and the original cluster centroids (*L_i*) were calculated. City size and cutoff value were 50 and 0.9, respectively for this scenario.

#### Scenario 4

The last scenario is designed with the same aim as scenario 3. The difference here is that we first define a null hypothesis and then build the null space. Using this null space, we can define a p-value for results from our proposed method. The null hypothesis here is that our proposed method either perform as “good” or “worse” when compared to a method in which time points are chosen randomly and then clustered (in contrast to our method where density is defined for each point and high-density points are clustered). To do this for each simulation, first the number of high-density points is calculated, and then random points are drawn from the same simulation 10,000 times (number of random samples drawn is the same as the number of high-density points). Next, these random points are used in clustering and the sum of the distance of resulting centroids from the original high-density location (*L_i*) is calculated. This results in 10,000 distance values. We then compared the sum of the distance resulting from our proposed method to these 10,000 values. The *p*-value is defined as the ratio of times the distance resulting from null method was lower than the distance resulting from our proposed method. This approach resulted in one *p*-value for each simulation. For each of the path spread values, the simulation was repeated 1,000 times. City size and cutoff value were 50 and 0.9 respectively for this scenario.

### Analysis of fMRI Data

The data used for this study has been published by our group previously (Damaraju et al., 2014). This data was acquired from 151 SZ patients and 163 HC participants. Resting state fMRI scans were acquired using 3T scanners at seven different sites. A gradient-echo planar imaging paradigm was used with the following parameters: FOV of 220 x 220, TR = 2 s, TE = 30 ms. 162 volumes were acquired during the scanning session. Table 2 shows some of the important parameters for both the data and the algorithm.

**TABLE 2:** Important parameters.

**Data parameters**	
Schizophrenia individuals	*N* = 151
Healthy control	*N* = 163
repetition time (TR)	2 s
Number of volumes	162 volumes
Algorithm parameters	
*d*_*j*,*t*_	Density for subject j at time t
*d*_*thr*_*j*_	Density threshold for subject j
N_*j*,*t*_	High density neighborhood for subject j at time t

In summary, for preprocessing, motion correction, slice-timing correction, despiking, registering to MNI template and smoothing was done. Prior to conducting gICA, each voxel time course was variance normalized. Then gICA was used as implemented in the GIFT software (Calhoun et al., 2001; Calhoun and Adali, 2012). First, the subject-specific time dimension was reduced from 162 to 100 using principle component analysis (PCA) approach. Then, all subjects’ data were concatenated and group level dimension reduction (using PCA) was used to reduce the dimension to 100. Next, using gICA, the data was separated into maximally independent spatial maps and their associated time series (Calhoun et al., 2001; Erhardt et al., 2011). Subject-specific time series and spatial maps were calculated using back reconstruction methods. For a full explanation on gICA please (see Allen et al., 2014).

All spatial maps were visually inspected and 47 were used as components of interest based on the literature findings. For a more complete explanation of the analysis for this data set (see Damaraju et al., 2014).Here, we used the same 47 components used in that study. Consequently, for each subject we are working in a 47-dimension space. As mentioned in the first part of section “Materials and Methods,” density was calculated for each time point of each subject and high-density points for all subjects were concatenated and k means was used to cluster this matrix (we have 47 features for k means). This analysis thus represents an analysis in a high dimensional (D = 47) space. The cluster centroid that we calculated using k means was used to cluster all the time points for all subjects (k means only clustered the high-density time points). After doing this last clustering, each subject time point belongs to one of the clusters and we can calculate several metrics based on these results.

The first metric we calculated was mean dwell time. This metric is the average time each subject spends in one specific cluster (i.e., how long each individual stays in a given cluster). The next metric is called the transition number and is simply the number of times each subject changes states. For the last metric, we calculated a transition matrix that is the number of times each subject changed from one specific state to another state (if we have five clusters, this will be a 5 by 5 matrix). All these metrics were compared between HC and SZ groups.

## Results

### Simulation

Figure 1 depicts one of the simulations where cluster size is 50 and 3 clusters are simulated therefore we have 150 time points all in all. This data set was simulated to have three high traffic nodes (i.e., location in our defined space that has a lot of points that are in their close neighborhood) and a path spread of 0.2. As seen in this figure, points closer to high traffic nodes have higher density and therefore can pass density thresholds, while points further away from these clusters (e.g., more noisy points) have lower density.

**FIGURE 2:**
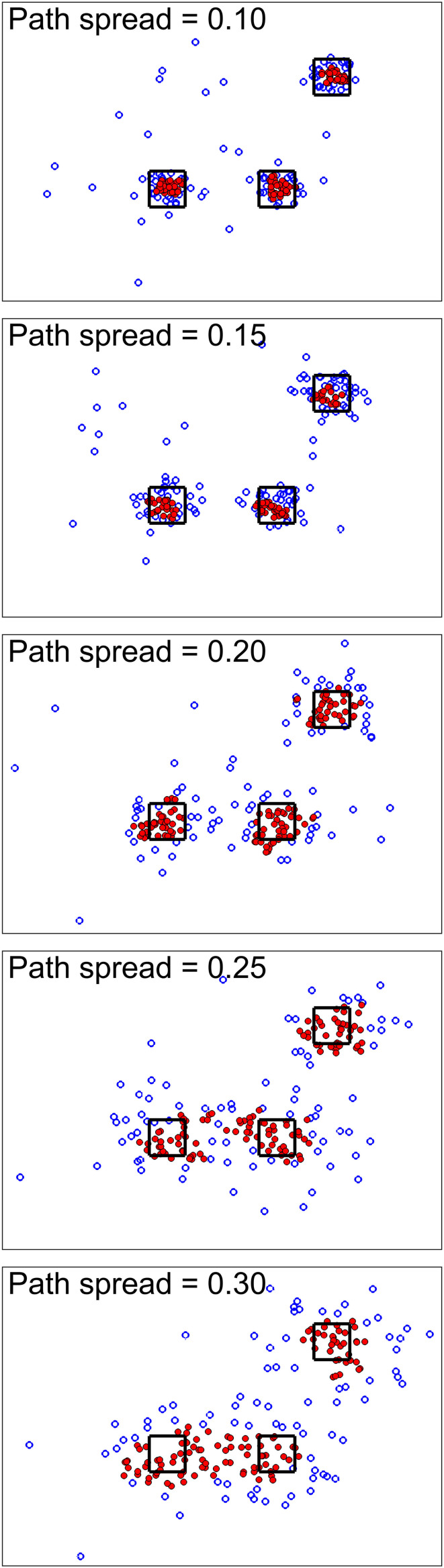
Simulation scenario 1. For this scenario, different simulations were done using different path spread values. Circle markers show points in our defined 2D space. The filled ones are high-density points while the empty ones are points with lower density values. The black squares are centered around the high traffic node (their size is for better visualization only). Higher path spread causes the clusters to have more overlap as was expected.

For the first simulation scenario, we wanted to show the effect of path spread parameters on the simulation. Figure 2 shows the simulation results for different path spreads. Here filled markers show high-density points while empty markers are points with lower density values. The black squares are the high-density neighborhood. As seen here, increasing path spread causes clusters to have more overlap with each other.

**FIGURE 3:**
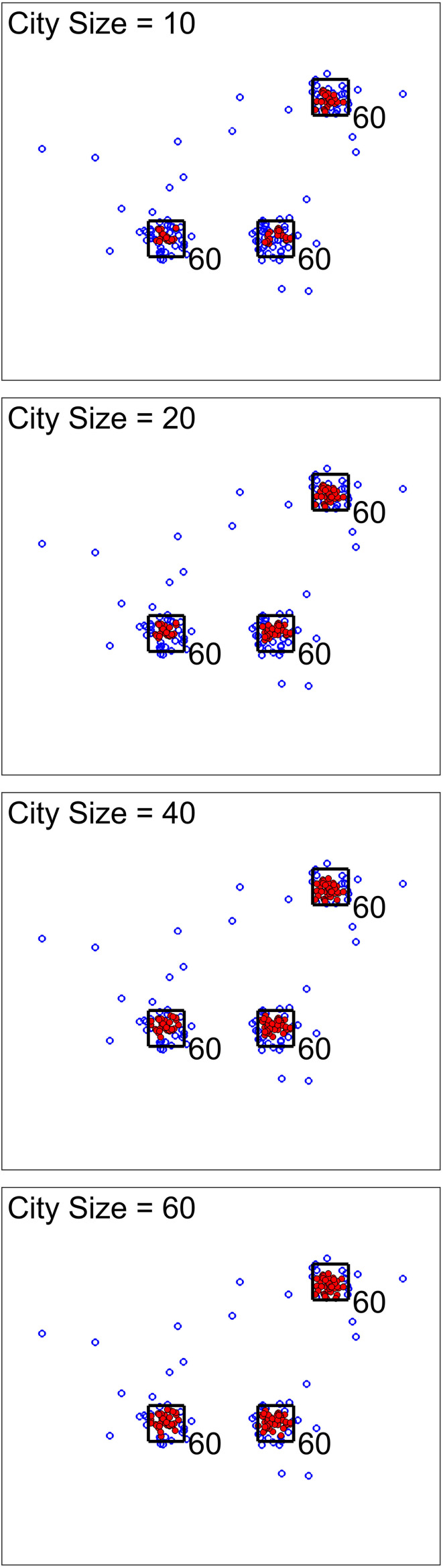
Scenario 2 results. This analysis shows one simulated data set with balanced clusters using different city size values. The black squares indicate the high traffic nodes used for simulation (the size is for visualization). Each marker shows one point of the simulated data in the 2D space. The filled markers are high-density points while the empty markers are low density ones. As expected, the city size parameters do not seem to significantly impact the results.

**FIGURE 4:**
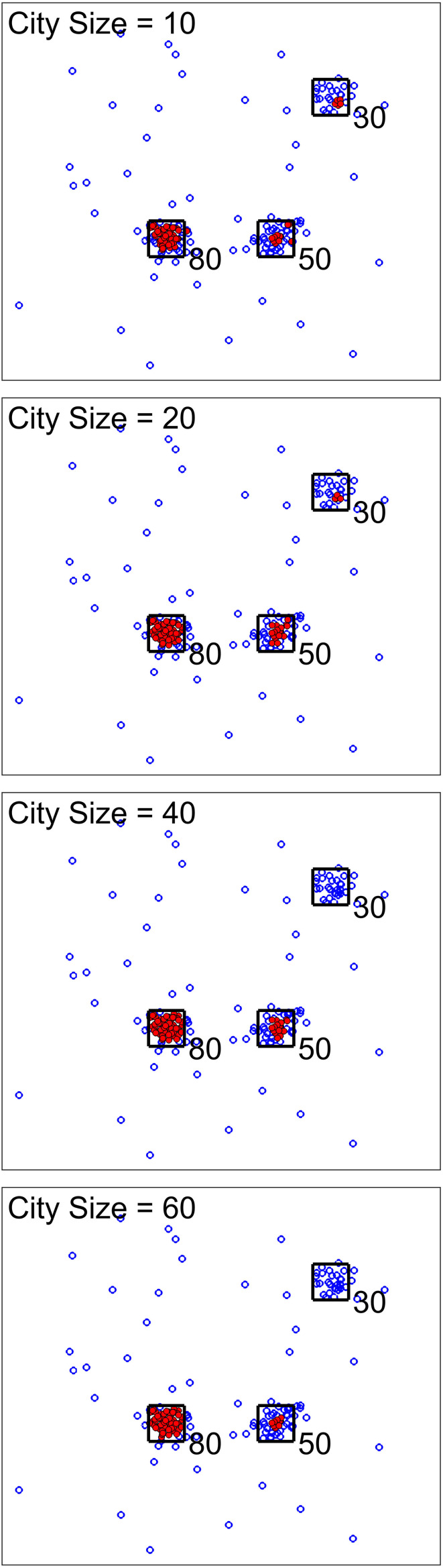
Scenario 2 results. We analysed one simulated data set with unbalanced clusters using different city size values. The black squares show the high traffic nodes used for simulation (the size is for visualization). Each marker shows one point of the simulated data in the 2D space. Filled markers are high-density points while the empty markers are low density ones. As seen here, larger clusters have more high-density points. In addition, using larger city sizes, we were unable to generate any high-density points for clustering with the 30 original members.

For the next scenario, our goal was to show the effect of city size (one of the analysis parameters). Figures 3, 4 show two cases for this scenario. For the first cases, the data set was simulated to have clusters with equal member numbers (60), while for the second case each cluster had a different number of members. Figure 3 shows the first case where clusters are balanced. As seen here, the points closer to the center of the squares are mostly high-density points. For all city sizes, we have an almost equal number of members from each cluster as high-density points. This is different for the second case (Figure 4). The clusters with a higher number of members (80) have more high-density points for all city size values. Please note that the smaller cluster (with 30 members) does not have any high-density points for city sizes 40 and 60 (which are larger values compared to 30). This is the reason we have added city size to our proposed approach. In contrast, if we would calculate the density of one point using all of the other points in the data, the larger clusters would dominate the results. Therefore, this city size parameter defines the cluster size detectable by the algorithm.

**FIGURE 5:**
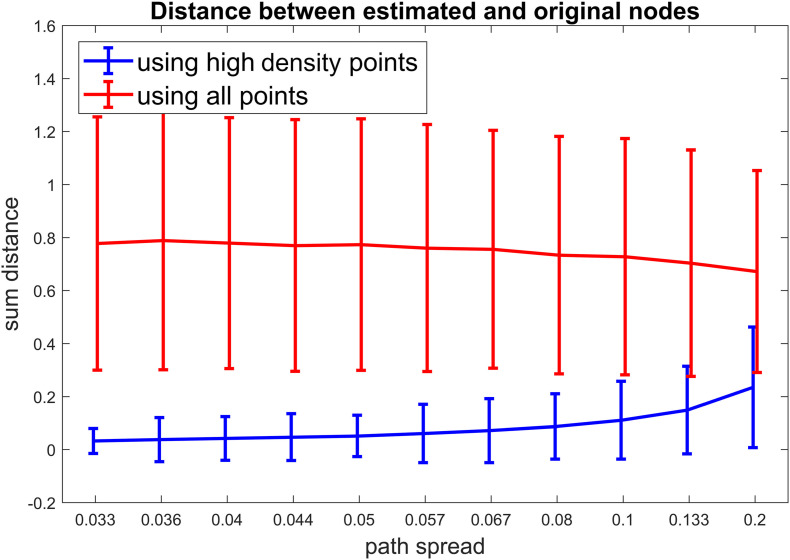
Scenario 3 results. For this simulation, we aimed to examine if our calculated metric improves the clustering accuracy. For each simulated time series, First all the points were included in the clustering (the red line). Next, only the high-density points were included in the clustering (the blue line). To access the accuracy of clustering, the distance between estimated cluster centroids and the true cluster centroids were calculated and summed for our three clusters. As visible in this figure, our proposed metric improves the accuracy of clustering by a noticable margin (especially for lower path spread values).

For scenario 3, we compared our proposed method to a method which uses all time points for clustering (In contrast to our approach which only uses high-density points). For different path spread values 1000 simulations were ran and the sum of distances from estimated clusters to the original clusters were calculated for each simulation. Figure 5 show the results for this scenario. It is obvious that our method has resulted in lower distances for all path spread values. This is more apparent for smaller path spreads. For this scenario around 50% of points passed the density threshold for each cluster. This value was not different between different path spread which means that no matter how much points spread around the cluster centroids around 50% of them are high density points. On the contrary, the number of high-density points for noisy points (points not in any given cluster) are below 5% of those points which is a positive finding as it shows that those points are not included in clustering part.

**FIGURE 6:**
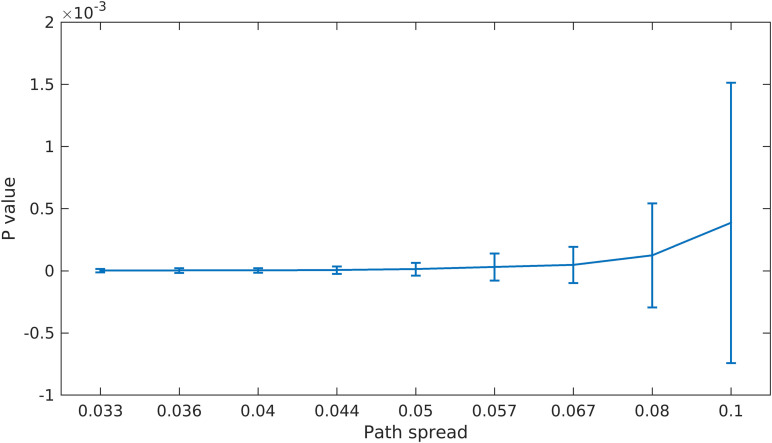
Scenario 4 Results. For this scenario, we first built a null space by using random points in clustering. We then calculate the *p* value by finding the portion of times the randomly selected points resulted in better clustering accuracy (defined as the sum of distances between estimated centroids and the true ones). As can be seen here, even for the largest path spread values the *p* value is small (compare to significant *p* value of 1 × 10^–2^).

As explained in the previous section, we built a null hypothesis for the last scenario to check if the proposed feature selection step works better than a completely random method. Figure 6 shows the *p* values calculated using this approach. The *p* value for all path spread values are low and also pass a 0.01 threshold (the average *p* value for the largest path spread is less than 0.5e-3). These results provide evidence that our feature selection step (finding points with high density) is informative and improves the results considerably.

### Real Data

**FIGURE 7:**
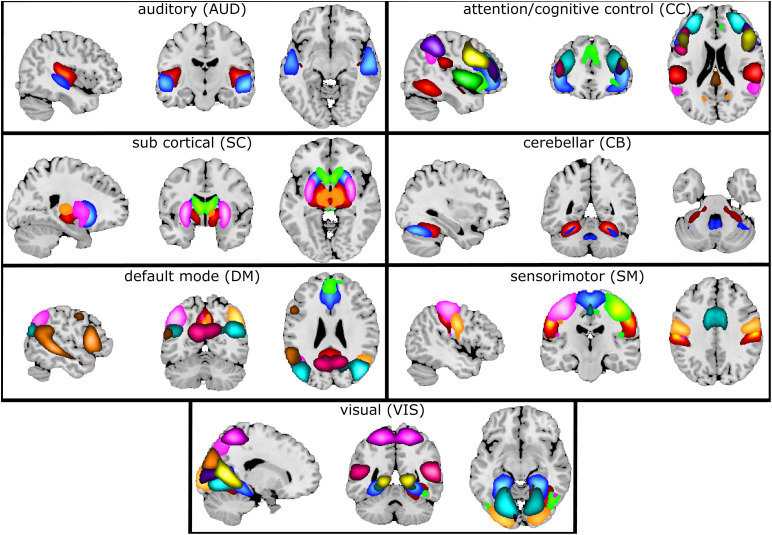
Composite maps of the seven connectivity domains. After gICA, 47 components were chosen and grouped into seven different connectivity domains. Each color in each domain represents a separate component.

Figure 7 illustrates the selected 47 components grouped into seven connectivity domains following our earlier work (Damaraju et al., 2014).

**FIGURE 8:**
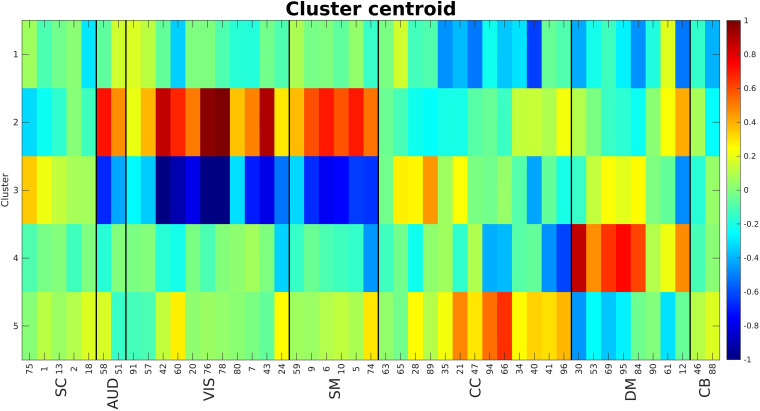
Cluster centroids. Cluster 1 shows a strong negative activation in CC and DM. Clusters 2 and 3 show strong (positive and negative respectively) activations in AUD, VIS and SM domains. Clusters 4 and 5 show strong activations in CC and DM. The main point here is these cluster centroids are different from each other while pairs 2–3 and 4–5 show very opposite patterns. The numbers at the bottom of the image are the component numbers.

The elbow criterion was used to determine a cluster number of 5. We used 0.9 as the cutoff for calculating density threshold based on the simulation results. City size was equal to 16 (10% of number of time points). Figure 8 shows the calculated centroids for the five clusters. The centroids (each representing one cluster) are visibly different between clusters. Cluster 1 shows negative activations for cognitive control (CC) and some components of default mode (DM). Clusters 2 and 3 show strong activations in auditory (AUD), visual (VIS), and sensorimotor (SM) domains with cluster 2 being mostly positive while cluster 3 is mostly negative. Clusters 4 and 5 show somewhat strong activations in CC and DM domains. One very interesting observation here is that cluster pairs 2–3 and 4–5 show opposite effects.

**FIGURE 9:**
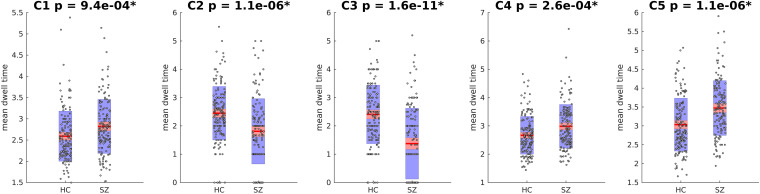
Mean Dwell Time for each Cluster. Mean dwell time is significantly different between HC and SZ for all clusters. SZ patients stay more in clusters 1, 4, and 5 that show strong activation in DM and CC (both positive and negative). HC subjects stay more in cluster 2 and 3 that have strong activations in AUD, VIS, and SM. Asterisk indicates *p* < 0.01 (*p*-values are corrected using FDR method).

**FIGURE 10:**
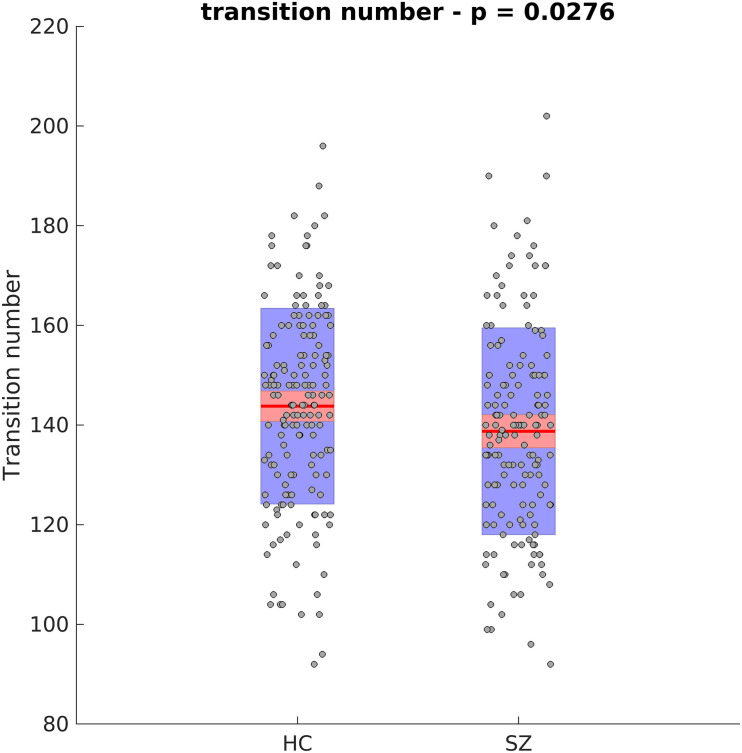
Transition Number Between Different Clusters. HC subjects move between different clusters more. Asterisk indicates *p* < 0.01.

Next, the cluster centroids were used to cluster all the original data. The first metric calculated based on these final clustering results, was the mean dwell time. Figure 9 show the mean dwell time for all 5 clusters. The *p*-values are corrected using FDR method. As seen, dwell time for all clusters are significantly different between HC and SZ subjects. SZ subjects tend to stay more in states 1, 4, and 5 (which show similar patterns where clusters 4 and 5 show opposite patterns). HC subjects tend to stay more in clusters 2 and 3. Figure 10 shows that the transition number between clusters is significantly higher in HC subjects compared to SZ (*P* < 0.05).

**FIGURE 11:**
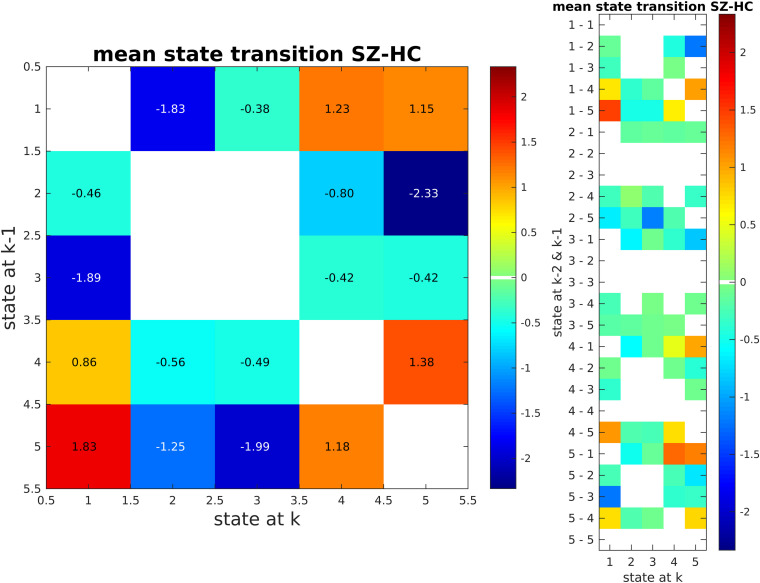
State Transition Matrix for Two Lag Values (SZ - HC). SZ subjects tend to transition between clusters 4 and 5. These are the clusters that SZ subjects tend to stay in more as compared to HC subjects. In contrast, HC subjects go from states 2 and 3 to states 4 and 5.

We also calculated state transition matrices for 1 lag. FDR was used to correct for multiple comparison (*p* < 0.01; Figure 11). Only significant entries for the matrix are shown. The values of the matrix are the mean state transition for SZ subtracted by the mean state transition for HC.

Results from different analysis parameter are show in Supplementary Figures 1–6.

## Discussion

In this article, a new approach is used to study the dynamic aspects of brain functional activity. This approach allows us to identify specific activation patterns exhibiting similar behavior by analyzing the trajectory of the dynamics directly, hence avoiding the need for windowing. First, we used a simulation to validate our approach. Then, using an actual dataset including SZ patients and HCs, we have demonstrated the utility of our approach.

In the null simulation, we show that our method works well for finding the high traffic locations. It is important to note that this simulation was done in 2D, while the data occupy a high dimension and should benefit even more from our proposed approach for selecting high-density time points.

In our simulation, we found that our approach does not work as well for medium noise amplitudes. To explain this, note that if the noise amplitude is relatively high, the affected points will have a higher distance from the original high-density node; therefore, their corresponding density will be lower. This can prevent those points from passing the high-density threshold, which results in their exclusion from clustering. This is an interesting aspect of our proposed method and might point to possible noise reduction applications that require further exploration.

Using a previously analyzed dataset, we demonstrated the use of our proposed method. Results show that the state transition number is significantly higher in HC subjects. More specifically, HC subjects tend to transition between activation states more frequently than the patients. In agreement with this finding, some studies have reported diminished connectivity dynamism in SZ compared to HC using different approaches (Miller et al., 2016; Mennigen et al., 2018b).

One of the state that SZ subjects tend to occupy more (i.e., state 1) shows an overall sparse activation. This is to some degree in line with the previous study that has used the same data set (Damaraju et al., 2014). In that study, the authors reported that SZ subjects stay in states that show weak connectivities in general. This sparse connectivity has been reported in other studies as well (Garrity et al., 2007; Whitfield-Gabrieli et al., 2009; Liu et al., 2012).

In the previous study (Damaraju et al., 2014), it was reported that HC subjects stay more in states that have strong connectivity in AUD, VIS, and SM. In our study, HC subjects have a significantly higher dwell time for states 2 and 3, which shows a strong connectivity in the same three domains. In addition, this observation can be viewed as more evidence for disconnectivity in SZ that has been previously reported. For a review of the matter (see Pettersson-Yeo et al., 2011).

Beyond this, our approach shows that SZ subjects transition more between states 4 and 5 when compared to HCs, while HC subjects tend to transit between states 2 and 3 to states 4 and 5 (Figure 11). That is, SZ subjects tend to stay in states that are similar to each other (strong connectivity in DMN and CC), while HC subjects switch between states that are quite different (i.e., transit between states 2, 3 to 4, 5). A restriction in dynamic range like what we found here has been reported in another study (Miller et al., 2016).

As can be seen from Figure 8, cluster pairs of 2 and 3 have an almost opposite spatial map. This can point to an oscillatory organization of brain networks. In a similar fashion, Gutierrez-Barragan et al. (2018) reported several functional states with opposing spatial maps. We have found this phenomena in our other work using the same dataset (Faghiri et al., 2021). In our future work, we want to explore if there is any difference between SZ and HC in their ability to oscillate between the opposite state pairs.

There are some limitations to our approach. First in our proposed formulas, we have one major parameter that we have to define (city size). As we showed using our simulations, City size determines the size of detectable states (in our defined high dimensional space). This parameter is quite similar to window size in sliding window approaches, where using large window sizes results in undetected information while using short window sizes increase the standard error of the estimator. For our approach, we performed our whole pipeline on fBIRN data set using different city size values and found no notable difference between the results. As a rule of thumb we suggest using 10 percent of time courses length as the city size.

Another limitation of the proposed method is that it does not directly consider the connectivity aspect of fMRI as in other studies (e.g., calculating a windowed Pearson correlation and then clustering). However, because of the nature of gICA, the concept of connectivity is indirectly present in our results. Each component resulting from gICA can be viewed as a small connectivity between smaller regions. In addition, this method uses all component information for the analysis; therefore, we are not able to use results to study a subset of the whole components. This could be done by rerunning analysis on the subsets of the components. This is a topic for future work. Finally, although the number of subjects in this study is relatively large for a clinical study, the impact of study size cannot be overlooked. Future studies are needed for both examining the replicability of the results and the impact of study size on this specific algorithm.

## Conclusion

Our method provides a new and useful approach for studying brain dynamics that analyzes the data in a high dimensional temporal space without requiring any windowing. Using this approach, we found several interesting new results related to schizophrenia. Importantly, we believe our approach provides a novel way to study brain dynamics by computing metrics directly from the high-dimensional space (in contrast to the sliding window Pearson correlation approach that looks at each of the component pairs separately). In addition, our proposed method can also be used to visualize fMRI data in low dimensions (2 or 3) while preserving interesting information.

## Data Availability Statement

The data analyzed in this study is subject to the following licenses/restrictions. Due to limitations imposed by the IRB we are unable to share the raw data, but it is possible to share the derived results. In addition, all the code used in this study will be shared upon direct request. Requests to access these datasets should be directed to AF, ashkanfa.shirazu@gmail.com.

## Ethics Statement

The participants provided their written informed consent to the institutional IRBs to participate in the study.

## Author Contributions

AF: conceptualization, methodology, software, visualization, and writing – original draft. ED: data curation, writing, reviewing, and editing. AB, JF, DM, SM, BM, GP, AP, JT, JV, and TV: investigation, writing, reviewing, and editing. VC: supervision, conceptualization, writing, reviewing, editing, and funding acquisition. All authors contributed to the article and approved the submitted version.

## Conflict of Interest

The authors declare that the research was conducted in the absence of any commercial or financial relationships that could be construed as a potential conflict of interest.
