# Supplementary material for: Brain Density Clustering Analysis: A New Approach to Brain Functional Dynamics
Source: Front Neurosci. 2021 Apr 13;15:621716. doi: 10.3389/fnins.2021.621716 (PMC8076753; doi:10.3389/fnins.2021.621716)
Supplement: Supplementary file 1 [file Table_1.DOCX]

Table S1. Coordination of peak activations of gICA component maps.

|  | **Coordinate** |
| --- | --- |
| **Sub-cortical (SC)** |  |
| IC 75 |  |
| Bi substantia nigra | –15, –15, –9 |
| IC 1 |  |
| R putamen | 24 12,–3 |
| L putamen | –24, 12, –6 |
| IC 13 |  |
| Bi caudate | –9, 18, 0 |
| IC 2 |  |
| R globus pallidum | 30, –6, 0 |
| L globus pallidum | –30, –6.3 |
| IC 18 |  |
| Bi thalamus | –9, –15, 3 |
| **Auditory (AUD)** |  |
| IC 58 |  |
| L Heschl's gyrus | –36, –30, 12 |
| R superior temporal gyrus | 51, –15, 3 |
| IC 51 |  |
| R middle temporal gyrus | 63, –15, –9 |
| L middle temporal gyrus | –60, –18, –6 |
| **Visual (VIS)** |  |
| IC 91 |  |
| R lingual gyrus | 27, –66, –6 |
| IC 57 |  |
| L parahippocampal gyrus | –24, –45, –12 |
| R parahippocampal gyrus | 30, –45, –12 |
| IC 42 |  |
| R middle temporal gyrus | 51, –69, 6 |
| L middle occipital gyrus | –45, –72, 6 |
| IC 60 |  |
| R precuneus | 30, –78, 33 |
| L cuneus | –27, –78, 27 |
| IC 20 |  |
| R Middle Frontal gyrus | –30, –93, –6 |
| L superior frontal gyrus | 30, –90, 5 |
| IC 76 |  |
| Left lingual gyrus | –9, –78, –6 |
| IC 78 |  |
| R cuneus | 3, –87, 21 |
| IC 80 |  |
| R middle temporal gyrus | 54, –51, 12 |
| L middle temporal gyrus | –54, –51, 9 |
| IC 7 |  |
| R cuneus | 3, –84, 6 |
| IC 43 |  |
| R calcarine gyrus | 15, –63, 9 |
| IC 24 |  |
| R Superior parietal lobule | –32, –88, –1 |
| **Somatomotor (SM)** |  |
| IC 59 |  |
| R postcentral gyrus | 63, –15, 27 |
| L postcentral gyrus | –60, –18, 33 |
| IC 9 |  |
| L medial frontal gyrus | 42, –21, 54 |
| IC 6 |  |
| Right postcentral gyrus | 42, –21, 54 |
| IC 10 |  |
| L precentral gyrus | –36, –24, 51 |
| IC 5 |  |
| R precentral gyrus | 54, –6, 27 |
| L precentral gyrus | –54, –9, 30 |
| IC 74 |  |
| L SMA | 0, 0, 48 |
| **Cognitive control (CC)** |  |
| IC 63 |  |
| L fusiform gyrus | –42, –57, –12 |
| R fusiform gyrus | 45, –54, –12 |
| IC 65 |  |
| R inferior frontal gyrus | 51, 39, 3 |
| L inferior frontal gyrus | –45, 39, 3 |
| IC 28 |  |
| R inferior frontal gyrus | 33, 24, –6 |
| L inferior frontal gyrus | –33, 24, –6 |
| IC 89 |  |
| L supramarginal gyrus | –54, –57, 36 |
| R supramarginal gyrus | 60, –51, 9 |
| IC 35 |  |
| L precuneus | –6, –72, 39 |
| R cingulate gyrus | 3, –27, 27 |
| IC 21 |  |
| R middle frontal gyrus | 33, 54, 12 |
| L superior frontal gyrus | –33, 45, 21 |
| IC 47 |  |
| Cingulate gyrus | 0, –36, 27 |
| IC 94 |  |
| R inferior parietal lobule | –42, –42, 45 |
| IC 66 |  |
| R inferior parietal lobule | 42, −45, 48 |
| IC 34 |  |
| R inferior frontal gyrus | 42, 9 30 |
| L middle frontal gyrus | –45, 12 30 |
| IC 40 |  |
| Precuneus | 0, –60, 48 |
| IC 41 |  |
| R insula | 45, –3, 0 |
| L insula | –45, 0, 3 |
| IC 96 |  |
| R inferior parietal loble | 57, –30, 24 |
| L inferior parietal loble | –60, –36, 24 |
| **Default-mode (DM)** |  |
| IC 30 |  |
| Precuneus | 0, –57, 33 |
| IC 53 |  |
| L anterior cingulate gyrus | –3, 48, 12 |
| IC 69 |  |
| R medial frontal gyrus | 3 2042, 45 |
| IC 95 |  |
| L angular gyrus | –48, –63, 42 |
| IC 84 |  |
| R angular gyrus | 51, –60, 39 |
| IC 90 |  |
| R angular gyrus | 45, –75, 30 |
| L superior occipital gyrus | –36, –81, 30 |
| IC 61 |  |
| L middle temporal gyrus | –57, –42, 0 |
| R inferior frontal gyrus | –54, 15 6 |
| IC 12 |  |
| L precuneus | –12, –57, 15 |
| **Cerebellar (CB)** |  |
| IC 46 |  |
| L culmen | –24, –42, –24 |
| R culmen | 27, –48, –24 |
| IC 88 |  |
| R declive | 30, –75, –24 |

| 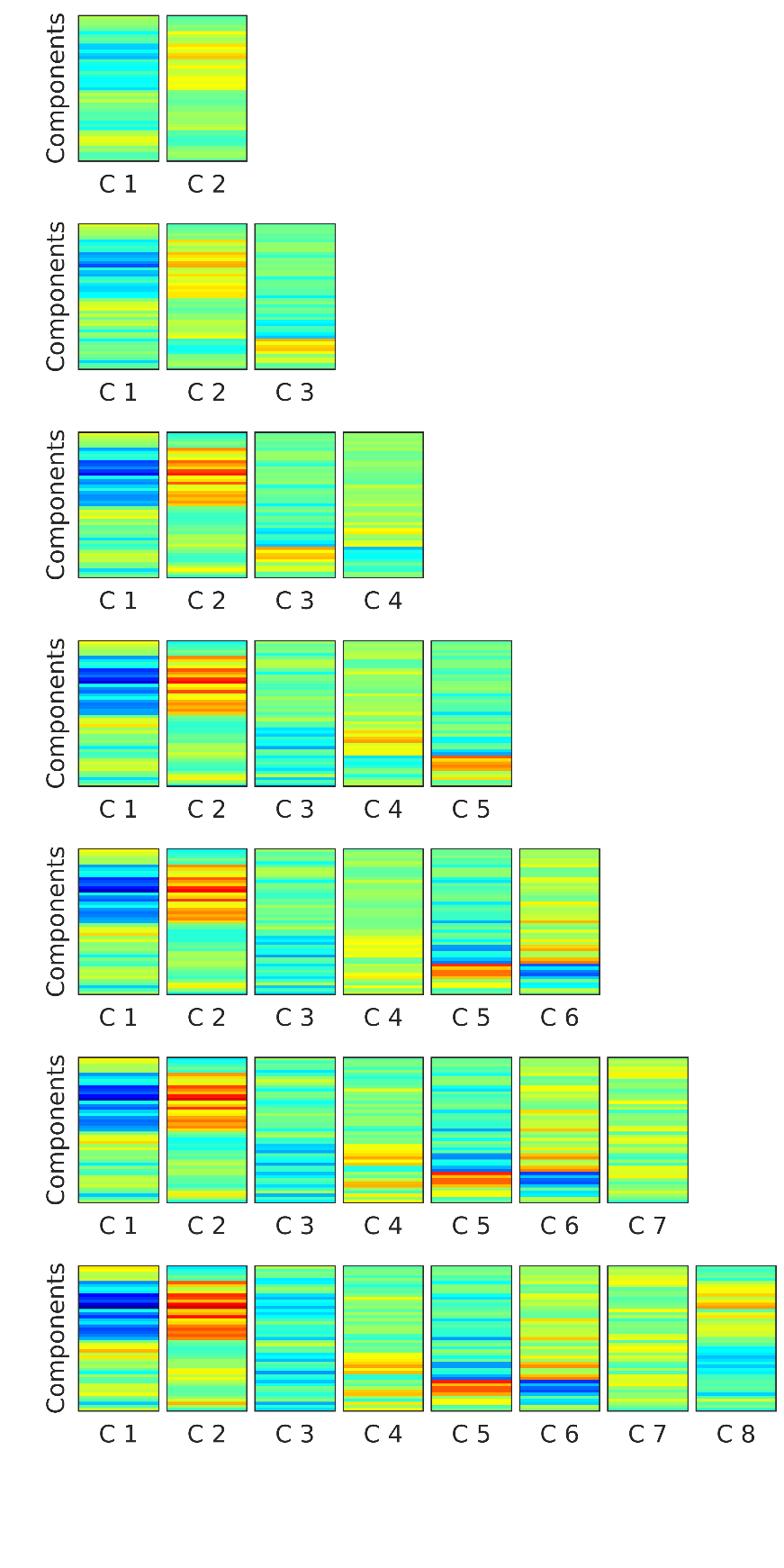  Figure S1. results from different cluster numbers. As can be seen as the number of clusters increases we see new clusters while the cluster visible in previous cluster numbers do not disappear. |
| --- |

| 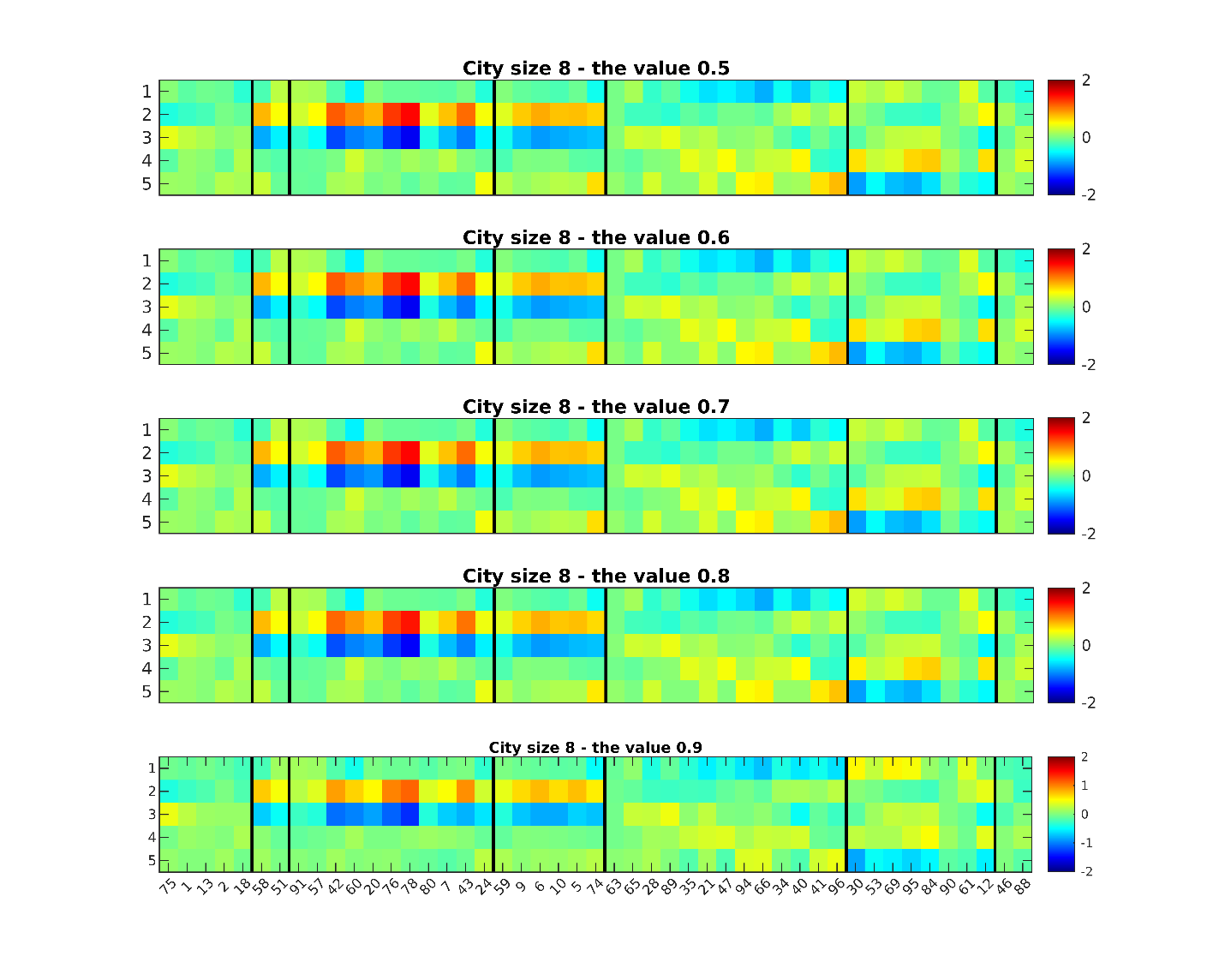  Figure S2. results from city size 8 and different energy threshold |
| --- |

| 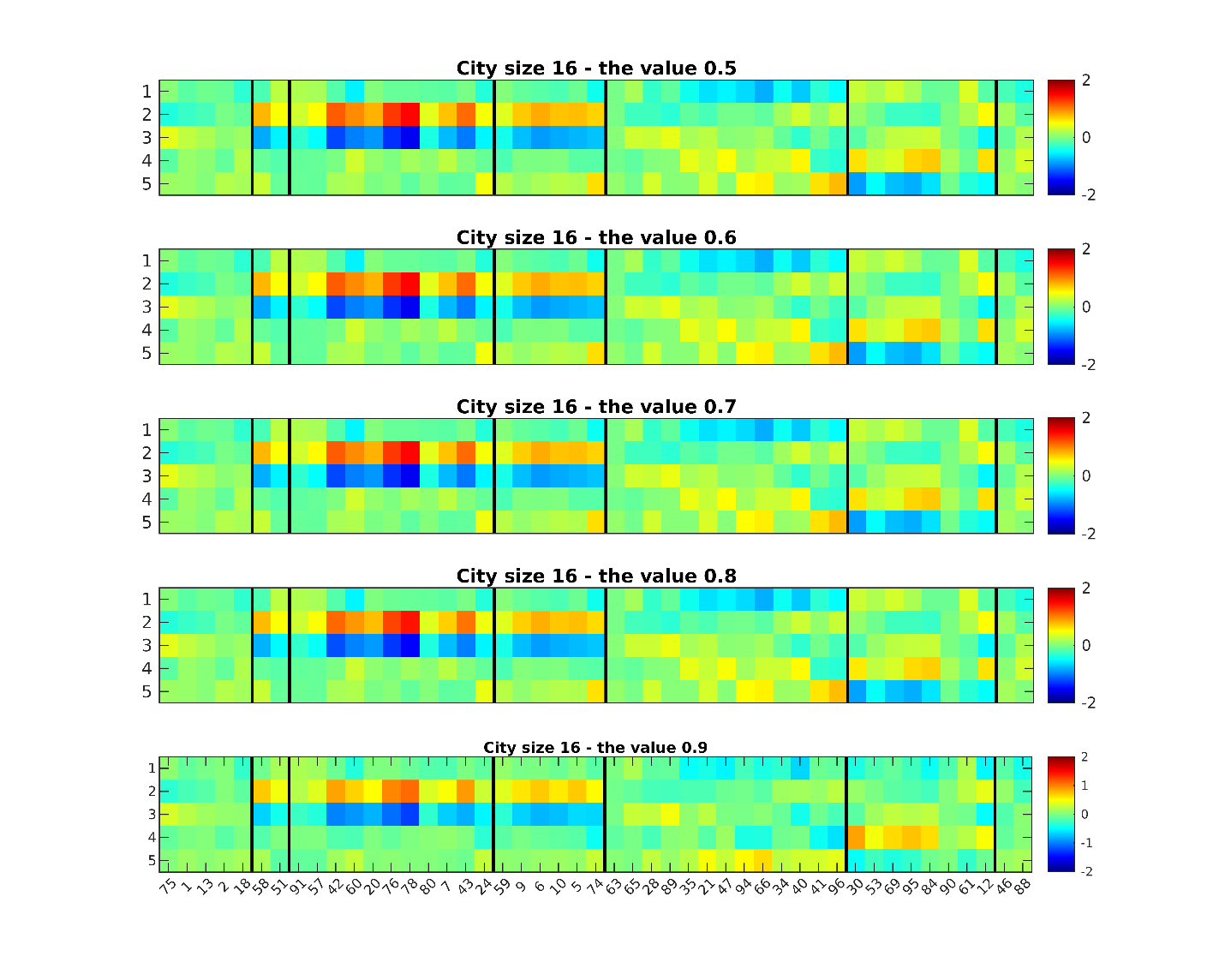  Figure S3 results from city size 16 and different energy threshold |
| --- |

| 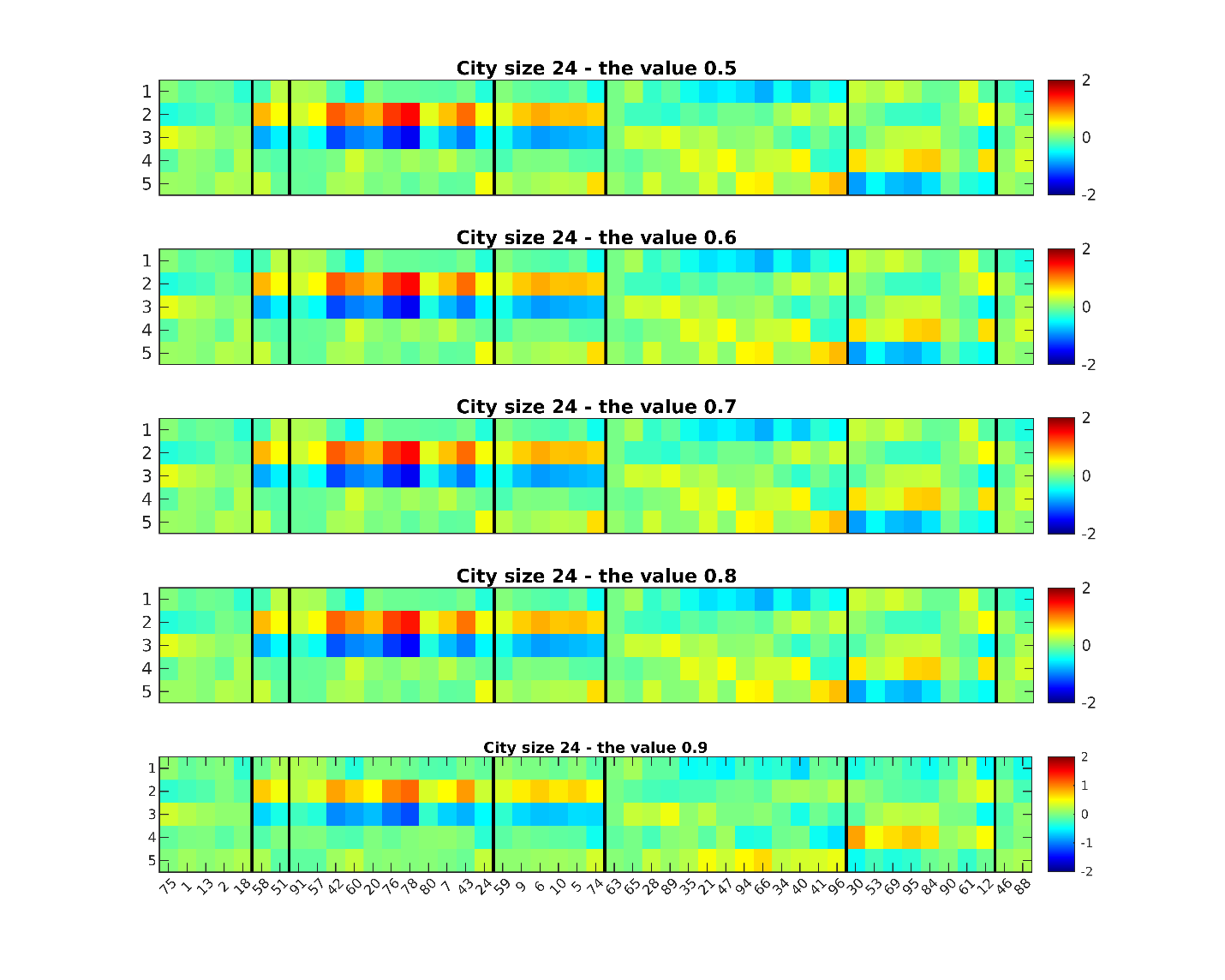  Figure S4 results from city size 24 and different energy threshold |
| --- |

| 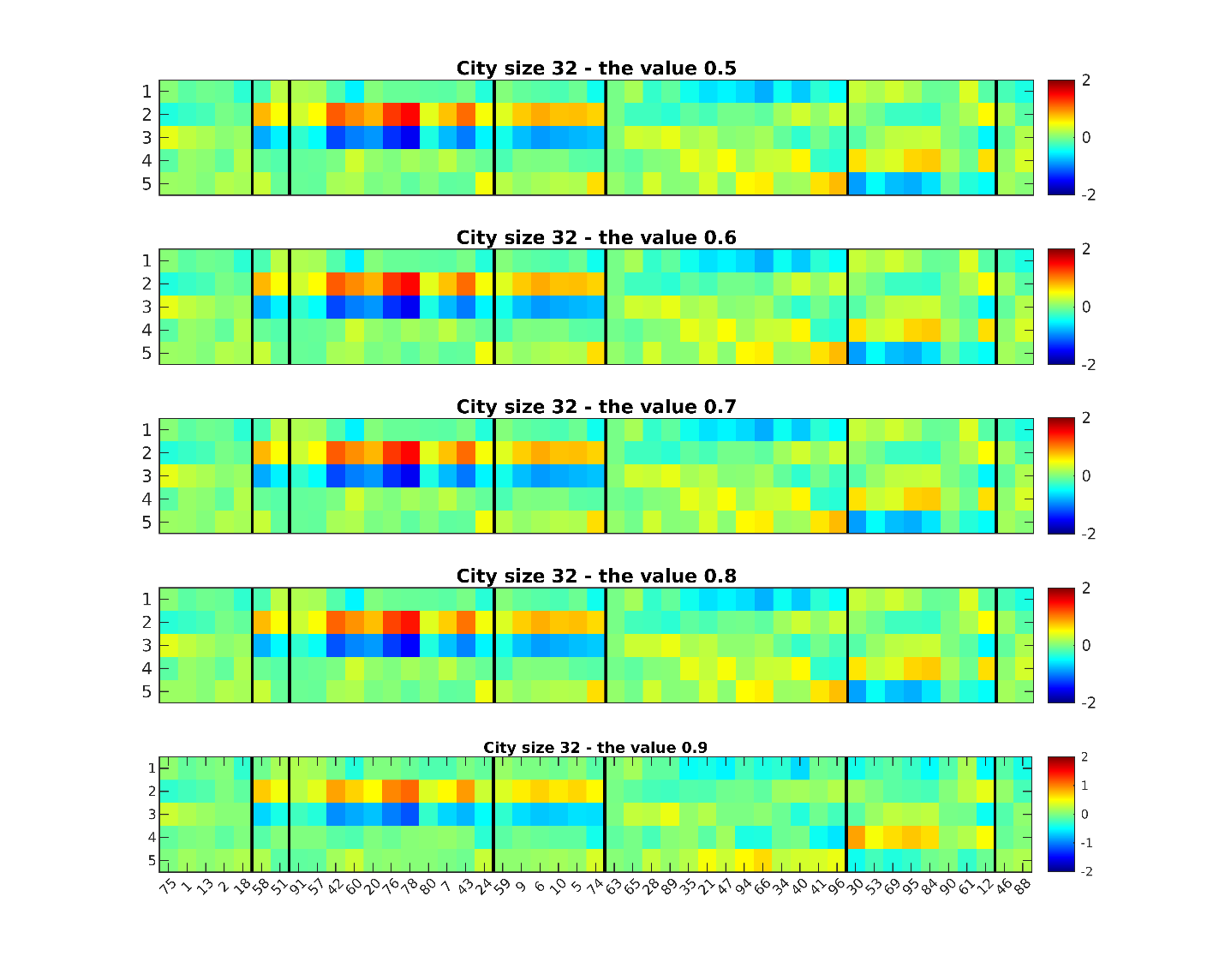  Figure S5 results from city size 32 and different energy threshold |
| --- |

| 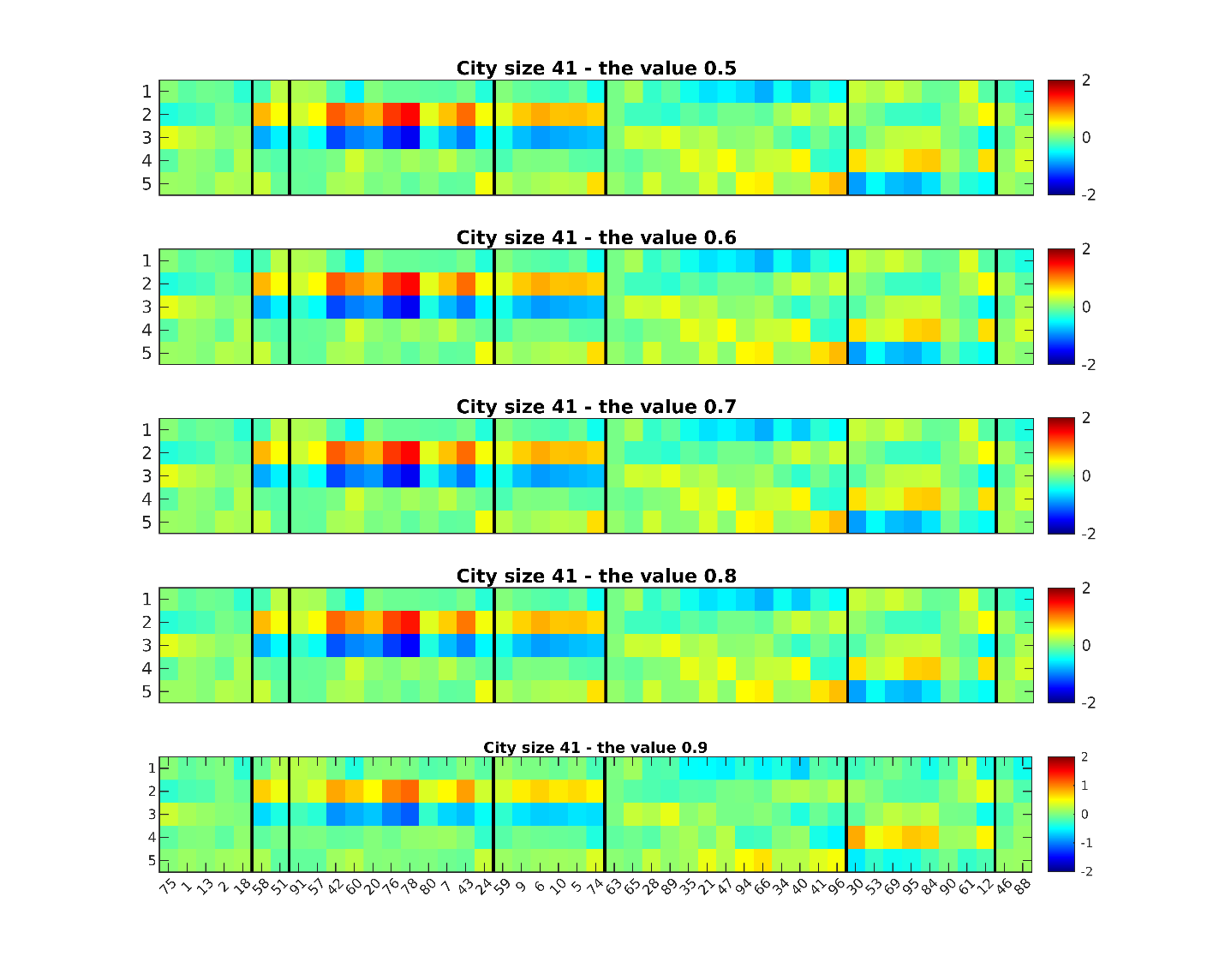  Figure S6 results from city size 41 and different energy threshold |
| --- |
